# A Quantitative Framework for Evaluating the Performance of Algorithm-Directed Whole-Population Remote Patient Monitoring: Tutorial for Type 1 Diabetes Care

**DOI:** 10.2196/72676

**Published:** 2026-03-25

**Authors:** Jamie Kurtzig, Ananta Addala, Franziska K Bishop, Paul Dupenloup, Johannes O Ferstad, Ramesh Johari, David M Maahs, Priya Prahalad, Dessi P Zaharieva, David Scheinker

**Affiliations:** 1 Division of Endocrinology, Department of Pediatrics, Stanford Medicine, 291 Campus Drive, Stanford, CA, 94305, United States, 1 (650) 7211300; 2 Stanford Diabetes Research Center, Stanford University, Stanford, CA, United States; 3 Department of Management Science and Engineering, Stanford University, Stanford, CA, United States; 4 Clinical Excellence Research Center, Stanford University, Stanford, CA, United States; 5 Department of Medicine, Stanford University School of Medicine, Stanford, CA, United States

**Keywords:** remote patient monitoring, continuous glucose monitoring, type 1 diabetes, chronic disease, precision medicine, quality improvement

## Abstract

Clinics continue to adopt care models shaped by the algorithmic analysis of continuous glucose monitoring (CGM) data, such as remote patient monitoring for type 1 diabetes. No clinic-facing quantitative framework currently exists to track the impact of such algorithm-directed care on patient outcomes and clinical workload. We used CGM data from the Teamwork, Targets, Technology, and Tight Control (4T) Study (Pilot n=135 and Study 1 n=133), in which algorithms enable precision, whole-population care by directing clinician attention to patients with deteriorating glucose management. Youth meeting criteria for clinical review are then contacted by Certified Diabetes Care and Education Specialists. Through iterative data analysis and meetings with a variety of stakeholders, we identified metrics for reviewing and revising clinical workloads, glucose management, and timeliness of care. For each metric, we developed an interactive dashboard to provide clinical and administrative leaders with an overview of the program. The metrics to track clinical workload were the total number of youths (1) in the program, (2) in each study, and (3) cared for by each clinician. The metrics to track glucose management were the number of youths meeting each criterion for review, including (4) total, (5) for each clinician, and (6) for each study. The metric to track timeliness of care was (7) the number of days since meeting criteria for clinical review. When presented at regular program leadership meetings, the metrics facilitated data-driven decision-making about clinical and operational components of the program. In this paper, we describe the process of developing and operationalizing this reproducible, clinician-facing key performance indicator tool to monitor an algorithm-enabled remote patient monitoring program. As the role of algorithms grows in directing clinical effort and prioritizing patients for care, this framework may help clinics track clinical workload, patient outcomes, and the timeliness of care.

## Introduction

Remote patient monitoring (RPM) programs for type 1 diabetes (T1D) care often use data provided by continuous glucose monitoring (CGM) systems [1,2]. RPM for T1D is shown to improve hemoglobin A_1c_ (HbA_1c_), quality of life, and glucose metrics in a variety of health care settings [3-5]. Numerous centers report the use of RPM for T1D care, and many include some level of algorithmic support in identifying patients for prioritization and clinical review or contact [6-10]. The growing use of CGM drives the demand for algorithms to manage data and inform care delivery, increasing the complexity of clinic operations [11,12]. Methods have been proposed for the retrospective analysis of the potential impact of changes to an algorithm that supports patient care, but there are few prospective approaches to monitoring the impact of algorithm-directed care [13].

Numerous studies report the use of an algorithm to inform patient care [6,10]. At the University of California, Davis, a single-center pilot study evaluated RPM for patients with newly diagnosed pediatric T1D [6]. The principal investigator reviewed each patient’s glucose data daily using the electronic health record (EHR) and Tidepool Data Platform (Tidepool Project). Monitoring began at hospital discharge and continued until the patient’s first outpatient clinic appointment. Patients were contacted as needed to adjust insulin doses based on their data. A statewide study in South Carolina provided RPM to underserved populations [10]. Research staff regularly reviewed patients’ glucose data and alerted the appropriate clinic if glucose values were abnormal. Research staff also generated regular reports identifying patients who either failed to transmit their glucose data or had high HbA_1c_ values. These reports were sent to appropriate local clinical teams, who were encouraged, but not required, to adjust patient treatment based on the data.

These, and other RPM programs, share common features that drive care provider workload, patient outcomes, and the cadence with which care providers engage with patients. Those measures are examples of key performance indicators (KPIs). KPIs track and can drive an organization’s progress toward strategic goals [14]. The potential of KPIs to monitor these aspects of care has been examined, but not in the context of RPM [14]. We sought to extend earlier findings and principles of the use of KPIs in health care to the setting of RPM.

In the Teamwork, Targets, Technology, and Tight Control (4T) Study 1 cohort, the mean HbA_1c_ was 6.58% at 12 months after diagnosis [9]. The Stanford 4T Study whole-population RPM tool, Timely Interventions for Diabetes Excellence (TIDE; Stanford researchers), uses CGM data to prioritize patients with T1D for personalized review and contact by a Certified Diabetes Care and Education Specialist (CDCES), a health care professional who educates and supports people with diabetes to improve health and reduce the risk of complications [15]. The TIDE algorithm informs glucose management, how patients are treated, and the regularity with which patients receive CDCES messages. The 4T Study developed a protocol within which CDCESs have safely and effectively adjusted insulin doses and increased patient engagement [16]. Within the 4T Study, CDCESs review patient data via the TIDE dashboard. They then interpret the algorithm-directed prioritization and patient data to recommend insulin dose adjustment and contact the patient via the EHR [16]. The 4T Study and the use of TIDE have been associated with significant, equitable improvements in glycemic outcomes, as well as reduced CDCES workload and burden [2,9,12,17].

To our knowledge, no clinician-facing quantitative framework is available to track how algorithm-directed care impacts clinical workload, patient glucose management, and timeliness of care. Such quantitative frameworks may be helpful for clinics that remotely access patient data, provide RPM-based care, and use algorithms to direct care delivery by, for example, identifying or prioritizing patients requiring care. This work aims to describe the process of developing and implementing KPIs and corresponding metrics to help clinicians, researchers, and administrators monitor clinical workload, patient outcomes, and timeliness of care.

## Methods

### The 4T Study

This work was conducted within the 4T Study. The 4T Study aims to improve care for newly diagnosed youth with T1D in Stanford’s Pediatric Endocrinology clinics. A summary of the 4T program is provided below, and details of the program have been published [9].

The 4T Study includes the 4T Pilot Study and 4T Study 1, as well as long-term follow-ups for each study. The long-term follow-up programs assess the ongoing impact of the 4T Pilot and Study 1 over an extended period. The 4T Pilot Study (n=135) enrolled participants from July 2018 to June 2020 [7,18]. 4T Pilot participants diagnosed before March 2019 were offered CGM, but RPM was not yet available. Those diagnosed between March 2019 and January 2020 were offered non–algorithm-enabled RPM (eg, Dexcom Clarity), which provided individual T1D insights rather than population-wide overviews such as TIDE. Pilot participants diagnosed during and after January 2020 were enrolled in the algorithm-enabled care platform for RPM. Participants diagnosed before January 2020 could transition to algorithm-enabled care when it launched. 4T Study 1 (n=133) enrolled participants from June 2020 to March 2022 and focused on initiating CGM use within the first 30 days after T1D diagnosis.

### Step 1: Identify an Appropriate Algorithm-Enabled Care Model

The quantitative RPM framework presented here is designed to support an algorithm-enabled care model. The 4T Study uses TIDE to support asynchronous review of CGM data for enrolled participants. All 4T participants had the choice to opt into TIDE when it launched.

Participants uploaded their CGM data via a personal smart device or study-provided iPod Touch (Apple Inc) to Dexcom Clarity. TIDE automatically pulled CGM data from the 4T Study’s Dexcom Clarity clinic account using a Python script, analyzed the data, and displayed results in an interactive data visualization dashboard created in Tableau (Salesforce). Guided by CGM clinical consensus metrics, the TIDE algorithm ranked participants based on the urgency of data review and contact by a CDCES [2,19,20].

Each week, the TIDE platform tracked whether participants met the following clinical categories: spending less than 65% of CGM time in range (TIR; 70‐180 mg/dL), more than 4% time below range (TBR) level 1 (<70 mg/dL), more than 1% TBR level 2 (<55 mg/dL), a drop in TIR of more than 15% points from the previous week, more than 50% missing CGM data, or meeting clinical targets [20]. Meeting targets refers to all participants who do not meet any of the other criteria (ie, they have >65% TIR, <4% TBR level 1, <1% TBR level 2, <15% point drop in TIR, and <50% missing CGM data). These clinical categories are based on clinical consensus guidelines [20]. Some metrics were updated between the Pilot Study and Study 1; for example, the TIR target was changed from 70% to 65%, changing the criteria flagged.

The CDCES team members used TIDE to view a list of participants, ranked by their clinical risk likelihood as defined by the CGM metrics listed above. CDCES team members then took appropriate follow-up steps if needed, such as sending secure messages with care recommendations to participants through the EHR. In both the 4T Pilot and Study 1, participant data were initially reviewed weekly for the first year after T1D diagnosis and monthly thereafter [9,12]. After the conclusion of each study, participants had the option to participate in an ongoing long-term follow-up program with monthly data reviews conducted by a CDCES.

### Step 2: Choose Key Performance Indicators

A metric is a quantitative measure of an aspect of a care process or the patient population. A KPI combines metrics into a high-level measure or visualization that reflects strategic goals and provides insights into the overall success and performance of an organization [14]. Commonly reported KPIs for health care organizations include patient waiting time, patient health status, patient quality of life, patient safety, and clinical efficiency [14].

Potential KPIs were eligible for inclusion if they aligned with the aims of the 4T Study and could be operationalized using patient CGM data within the existing 4T and TIDE infrastructure.

Using the above evidence-based list of recommended KPIs, inclusion criteria, and input from 4T clinicians, we selected clinical workload, glucose management, and timeliness of care for inclusion in the KPI framework.

We did not include safety or quality-of-life KPIs, as these are the focus of dedicated retrospective analyses (rather than ongoing KPIs). The 4T Study improved HbA_1c_, TIR, and patient-reported outcomes (PROs) with minimal TBR (<70 mg/dL) as published [7,9,17,21,22]. The algorithm does not recommend any specific insulin dose adjustment (that is done by the CDCES team) but rather alerts the CDCES that the CGM glucose data require attention.

### Step 3: Propose Initial Metrics for Each Key Performance Indicator

In 2022, the engineering team began developing a standardized approach to monitor the clinical workload, patient glucose management, and timeliness of care associated with the use of TIDE. Numerous ad hoc metrics were developed and measured based on the experiences of the 4T Study team, CDCES team, and administrative team [2].

The metrics were calculated from data continuously pulled from servers on each participant’s data completeness, the study cohort they were enrolled in, the most recent week they were shown in the TIDE dashboard, their CGM data, and the CDCES responsible for reviewing their data.

### Step 4: Develop Visualizations for Each Metric

We developed an instantiation of the quantitative framework with clinic-specific metrics deployed in an interactive dashboard engineered to streamline the monitoring and visualization of the entire program. All visualizations developed in Tableau were double-checked with manual calculations. We note that participants were enrolled in and departed from the 4T Study continuously; thus, the number of participants shown at any time in the visualizations is less than the total study population.

### Step 5: Iterate and Obtain Feedback

#### Feedback Sessions

At regular TIDE meetings, engineers presented the KPI framework and requested feedback. These meetings evaluated the perceived utility of this framework by collecting qualitative insights into the team’s firsthand experiences, perceptions, and suggestions regarding the framework’s implementation and impact. The meetings represented a diverse range of perspectives, currently including 5 CDCESs, 4 physicians, 4 engineers, 4 biostatisticians, 3 clinical research coordinators, as well as a staff scientist, exercise physiologist, postdoctoral fellow, and psychologist. All team members were encouraged to provide feedback.

Based on the feedback, inappropriate metrics were discarded, new metrics were suggested, visualizations were selected, chosen metrics and visualizations were refined, and actions were taken based on insights from the framework. Feedback was implemented when the team reached a consensus on a metric, visualization, or decision.

There were 10 TIDE meetings where engineers received feedback on the KPI framework. These semistructured meetings each lasted approximately 15 minutes and were conducted via Zoom (Zoom Video Communications). Questions followed a standardized interview guide (Section 1 in Multimedia Appendix 1) and focused on metric interpretability, usefulness, workflow fit, and decision-making impact. The engineering team took detailed notes (excluding any protected patient information) and sent the notes back to meeting participants for confirmation. We synthesized and grouped feedback by metric and visualization, noting discussion points, decisions, action items, and new ideas.

Based on the TIDE meetings, 2 smaller, more specific groups were directly interviewed to expand on and ensure feedback. These as-needed interviews lasted 30 minutes to an hour and were conducted via Zoom. Questions followed the same interview guide as the TIDE meetings (Section 1 in Multimedia Appendix 1), but more emphasis was placed on the specific team’s or individual’s expertise.

#### Select Metrics and Visualizations

Metrics and visualizations were discarded based on consensus from TIDE meetings. Metrics and visualizations were retained if (1) high and low values were readily understood by physicians and CDCESs, (2) they supported at least one clinical or operational decision, (3) they could be automatically computed using existing data and workflows, and (4) they provided unique information not given by any other retained metric. Metrics and visualizations were discarded if they failed one or more criteria. The clinical team assessed interpretability and actionability, and the engineering team evaluated feasibility. Everyone could comment on redundancy.

Throughout our process, 6 metric categories were discarded (Table 1). The first category of discarded metrics included measures of the number of CDCES messages and contacts through the EHR, which included metrics such as overall message count, contact count, TIR by message count, CGM time worn by message count, patients contacted versus suggested for contact, and patient response rate to clinician messages. These message and contact-related metrics were discarded due to a mismatch with our current workflow. CDCES messages are written and received within the EHR (Epic), while the KPI dashboard collects data from the TIDE dashboard. TIDE does not integrate or connect with the EHR. Additionally, CDCESs may not be able to act on insights from these metrics (ie, sending more messages or following up more) because of their limited time and availability.

**Table 1.** Excluded metrics and why they were excluded.

Metric	Reasons for exclusion
Number of CDCES^a^ electronic health record messages and contacts per participant	Does not integrate with current workflow May not drive decisions due to limited clinician time
Diabetes duration stratified by clinical categories and TIR^b^	Does not drive clinical decisions
Average glucose stratified by CDCES and study (or population of interest)	Does not provide any additional insights or drive additional decisions
Patient and family satisfaction ratings over time	Does not integrate with current workflow 4T Study has already retrospectively investigated this
Patient safety: number of adverse events by CDCES and study (or population of interest)	4T Study has already retrospectively investigated this
Clinical workload: CDCES time spent on RPM	Does not integrate with current workflow

a CDCES: Certified Diabetes Care and Education Specialist.

b TIR: time in range.

Second were measures of how diabetes duration may impact the clinical categories and CGM metrics, as well as the average diabetes duration for each of the CDCES’s patients. These were ultimately excluded since they would not drive day-to-day clinical or operational decisions and would be more appropriate for investigation in retrospective analyses.

Third were measures of participants’ average glucose. These were excluded since TIR is highly correlated and considered a more comprehensive measure [20,23].

Fourth, we did not include patient and family satisfaction metrics in the current KPI framework due to the lack of workflow integration. Currently, PROs are collected using REDCap (Research Electronic Data Capture)-based surveys, while the KPI dashboard uses information from the TIDE dashboard. Additionally, several 4T papers have already demonstrated positive PROs [8,22,24,25].

Fifth, we did not include safety measures because previous publications have shown that the 4T Study is associated with lower HbA_1c_ values, improved TIR, minimal TBR, and no unexpected serious adverse events [7,9,17,18].

Finally, we did not include an objective measure of CDCES time in the TIDE dashboard. Prior self-reported data from CDCES indicate approximately 15 minutes per complete patient review and an approximately 60% drop in time per patient per week with TIDE use (from 3.2 to 1.3 minutes) [2,26]. However, because TIDE’s host platform (Tableau) lacks session-duration logging, and CDCESs juggle other tasks, objective timing does not yet fit into the workflow. In the interim, the number of patients cared for by each CDCES is a useful proxy of workload. We may supplement the current proxy with CDCES time spent in the Epic EHR. Epic Signal logs task-specific activity (eg, chart review and patient messages), and these data can feed into the KPI dashboard via an application programming interface.

#### Refine Chosen Metrics and Visualizations

The next stage was an analytical approach to refine the chosen metrics based on an analysis of the data generated and the structured TIDE meetings with the research and clinical staff. In meetings, the stated goals of the process were to establish a more robust and comprehensive set of metrics for monitoring clinical workload, patient glucose management, and timeliness of care in the presence of algorithm-directed care. Interview questions and additional details are provided in Section 1 in Multimedia Appendix 1.

The above process of developing, evaluating, and soliciting feedback (steps 2‐5) was used iteratively to develop and refine KPIs and metrics based on these KPIs.

To monitor clinical workload in the 4T Study, the following metrics were identified: (1) the number of youths in the 4T program, (2) in each study, and (3) cared for by each CDCES. For tracking participant glucose management, the metrics include the number of youths meeting each criterion for clinical review, both (4) overall and for each (5) CDCES and (6) study. Initially, the metric for tracking the timeliness of care was the number of days since each participant’s last day meeting criteria for clinical review. This was later revised to (7) include only participants who have not been seen for more than 20 days and rank in the top 25% of patients with the longest time since meeting criteria. The detailed definitions of each metric are provided in Section 2 in Multimedia Appendix 1.

To visualize each included metric related to clinical workload, we used different colors to represent the number of patients requiring review, meeting targets, and missing >50% CGM data. For each of those 3 criteria, this was reported as the number of distinct patient IDs.

In the visualization of metrics related to patient outcomes, we used different colors to represent the number of patients in each clinical category. The clinical categories are less than 65% TIR, more than 4% TBR level 1, more than 1% TBR level 2, a drop of more than 15 percentage points in TIR, and less than 50% CGM wear time. For each clinical category, this is defined as the number of distinct patient IDs of participants flagged.

For the visualization of the metric related to timeliness of care, we displayed participant information only if more than 20 days had passed since the participant was last shown in the TIDE dashboard, and the participant was in the highest quartile for delayed care. The threshold of 20 days was selected based on a retrospective analysis of historical TIDE data, which revealed that the 75th percentile of time between participant appearances in the TIDE dashboard was 20 days.

For all metrics and visualizations presented, gaps or valleys in the data occur when, primarily due to the cadence of reviews, fewer CDCESs used TIDE during that period.

### Step 6: Use the Metrics to Drive Decisions

Feedback from all care team members highlighted 7 metrics as essential for monitoring clinical workload, patient glucose management, and timeliness of care (Table 2). Each metric is associated with a KPI displayed in an interactive figure with various filters and categorizations (Table 2).

**Table 2.** Included metrics, the corresponding key performance indicators, and the decisions the metrics drive.

Metric	Corresponding KPI^a^	Decisions the metric drives
Number of participants in the RPM^b^ program	Clinical workload	Modifying recruitment efforts or stringency of the criteria for participant review
Number of participants reviewed by each CDCES^c^ in the program	Clinical workload	Redistributing participants between the CDCESs
Number of participants in the RPM program for each study (or population of interest)	Clinical workload	Modifying recruitment efforts
Number of participants in the RPM program per clinical category	Patient glucose management	Hosting a session for CDCESs to teach their patients how to prevent low and high blood sugars or improve CGM^d^ connectivity
Number of participants in the RPM program per clinical category and reviewing CDCES	Patient glucose management	Evaluating the effectiveness of a CDCES’s new initiative
Number of participants in the RPM program per clinical category and study (or population of interest)	Patient glucose management	Identifying health disparities Changing the review frequency of a specific participant population
Number of days since participants’ most recent date of being displayed in the RPM program	Timeliness of care	Contacting a participant that has not been flagged for review recently

a KPI: key performance indicator.

b RPM: remote patient monitoring.

c CDCES: Certified Diabetes Care and Education Specialist.

d CGM: continuous glucose monitoring.

**Figure 1.**
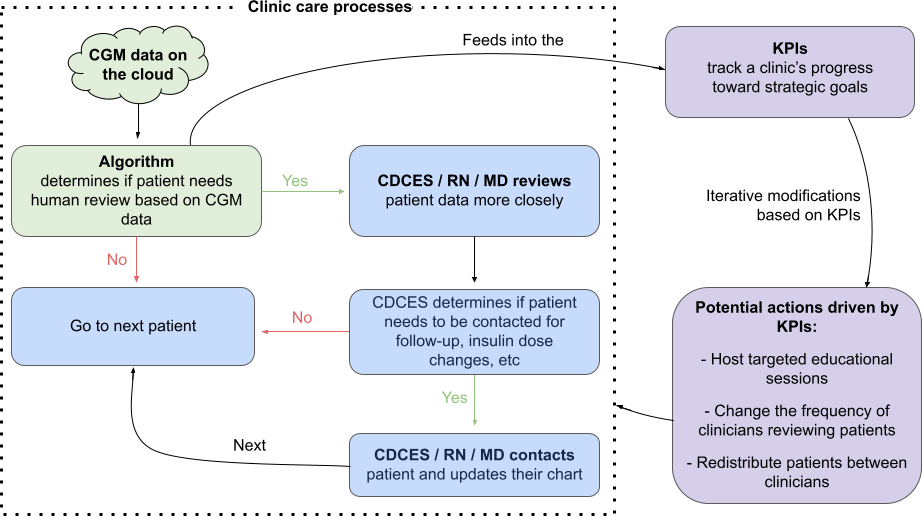
Care model and framework. CDCES: Certified Diabetes Care and Education Specialist; CGM: continuous glucose monitoring; KPI: key performance indicator; MD: medical doctor; RN: registered nurse.

At regular TIDE team meetings with clinical and administrative 4T team members, the dashboard was presented, and the metrics were discussed to facilitate an overview of the program. The KPIs were developed and used in parallel with the algorithm-enabled clinical care model and any related clinical studies. While the clinic provides patient care using the algorithm, the KPIs are based on the data gathered, are reviewed at a cadence set by the clinic, and may be used to adjust the care model or workflows (Figure 1). For key insights informed by each metric, refer to Section 3 in Multimedia Appendix 1.

Figure 1 shows how clinical care processes and the KPI framework interact. As noted in Step 1, the RPM algorithm ranks patients based on how urgently they need care. A CDCES then reviews patient CGM data and messages them to provide recommendations and education. As discussed in Step 3, the KPI dashboard pulls information from the RPM algorithm. The KPI framework’s metrics and associated visualizations help inform clinical and operational decisions and actions that modify the clinical care process.

### Ethical Considerations

The study was approved by Stanford’s Institutional Review Board (ClinicalTrials.gov: NCT03968055 and NCT04336969; IRB Protocol 52812). Patients and families who consented were enrolled in the 4T Study. Those who did not consent received standard clinical care, which allowed early CGM initiation but did not include RPM. Participants aged 18 years or older or legal guardians of minors provided informed consent, while youth aged 7 years or older provided assent prior to study initiation. Participants in the 4T Study were between the ages of 6 months and 21 years and started on CGM (Dexcom G6; Dexcom Inc) within the first month of T1D diagnosis. Youth and families were not reimbursed for their participation in TIDE, but they could receive compensation for completing PRO surveys, doing home HbA_1c_ fingerstick tests, participating in focus groups, and participating in the 4T exercise substudy. 4T participants also received a small monetary gift (US $10) for their birthdays as a thank you for their continued participation.

## Results

### Participants

Of the 268 participants in the 4T Pilot and Study 1, a total of 222 were eligible for RPM and were included in the KPI framework (Table 3). All 4T Study 1 participants (n=133) were enrolled in TIDE and included in the KPI framework. In the Pilot Study, participants diagnosed between July 2018 and February 2019 (n=46) were not enrolled in TIDE or included in the KPI framework, and the other 89 were included [7]. The 4T Pilot Study and 4T Study 1 enrolled 92% and 84%, respectively, of all newly diagnosed patients seen in our clinic.

**Table 3.** Participant demographics for the 4T Pilot and 4T Study 1.

Characteristics	4T Pilot	4T Study 1
Study length	2018-2020	2020-2022
Participants, n (%)	135 (50.4)	133 (49.6)
Participants on TIDE^a^, n (%)	89 (65.9)	133 (100)
Age (years), median (IQR)	10 (7-13)	11 (6-14)
Gender, n (%)		
Women	64 (47.4)	59 (44.4)
Men	71 (52.6)	74 (55.6)
Self-identified race and ethnicity, n (%)		
Non-Hispanic White	53 (39.3)	52 (39.1)
Non-Hispanic Black	0 (0)	1 (0.8)
Hispanic	29 (21.5)	49 (36.8)
Asian or Pacific Islander	19 (14.1)	11 (8.3)
American Indian or Alaska Native	0 (0)	0 (0)
Other	19 (14.1)	17 (12.8)
Not stated	15 (11.1)	3 (2.3)
Insurance type, n (%)		
Private	104 (77.0)	83 (62.4)
Public	31 (23.0)	47 (35.3)
Both	0 (0)	2 (1.5)
No insurance	0 (0)	1 (0.8)
Primary language, n (%)		
English	117 (86.7)	112 (84.2)
Non-English	18 (13.3)	21 (15.8)

a TIDE: Timely Interventions for Diabetes Excellence.

Unlike in traditional care models, there is no fixed number of participant counts. Participant counts change with glucose management, patients enrolling into the program, and patients transferring to other clinics, as well as the variable availability of the care providers, a format that has become more common in microrandomized trials [27]. Rigorous mathematical modeling to understand this is underway [28]. In visualizations for metrics 1-6, the gray area represents the duration of the 4T Pilot, and the white area represents the ongoing duration of the 4T Pilot long-term follow-up program.

### Metrics and Visualizations for Clinical Workload

**Figure 2.**
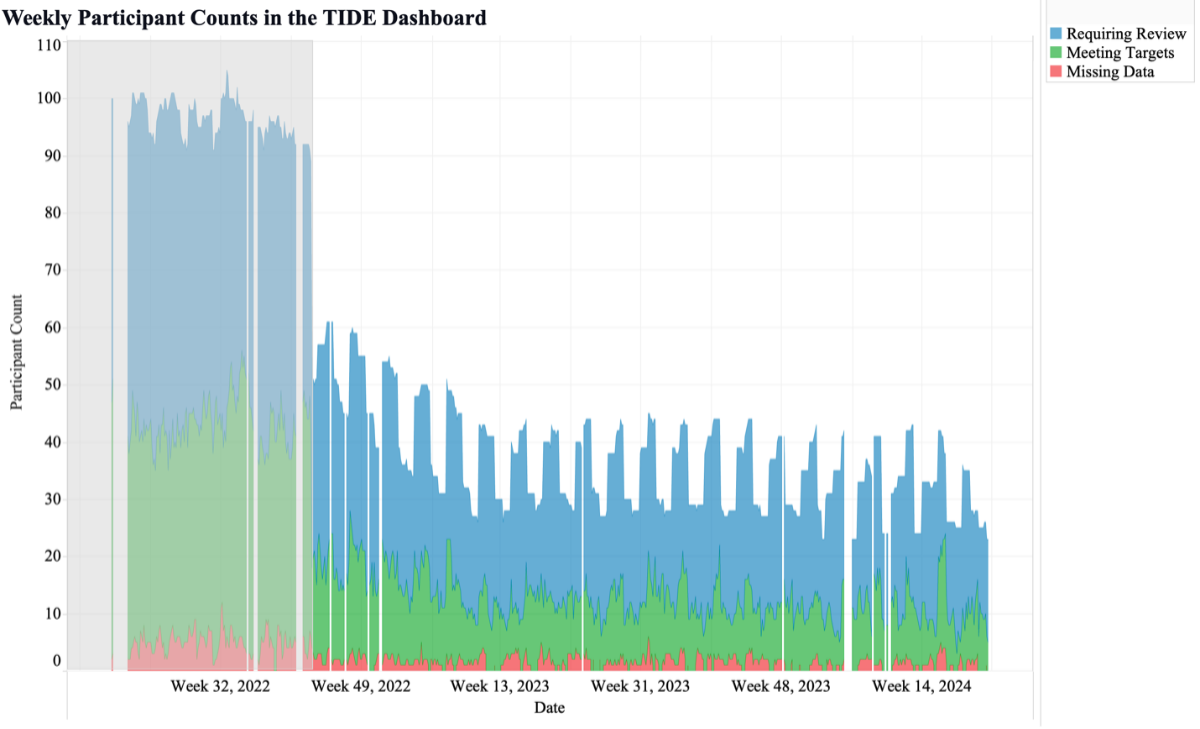
Weekly participant counts in the Timely Interventions for Diabetes Excellence dashboard, categorized by participants requiring review (blue), meeting targets (green), and with more than 50% missing data (red). TIDE: Timely Interventions for Diabetes Excellence.

The first metric was the total number of participants shown in the TIDE dashboard. By monitoring the number of participants shown in TIDE, this metric allowed for the tracking of population statistics over time. The visualization of the metric selected by the CDCES team included the total number of participants in the program over time, segmented by those who did and did not require review (Figure 2). Based on this metric and visualization, the 4T Study team could investigate recruitment efforts or participant withdrawal rates if the metric was unexpectedly low or high. This metric was also used to understand the stringency of the criteria for participant review if the number of participants being reviewed was too high or too low.

**Figure 3.**
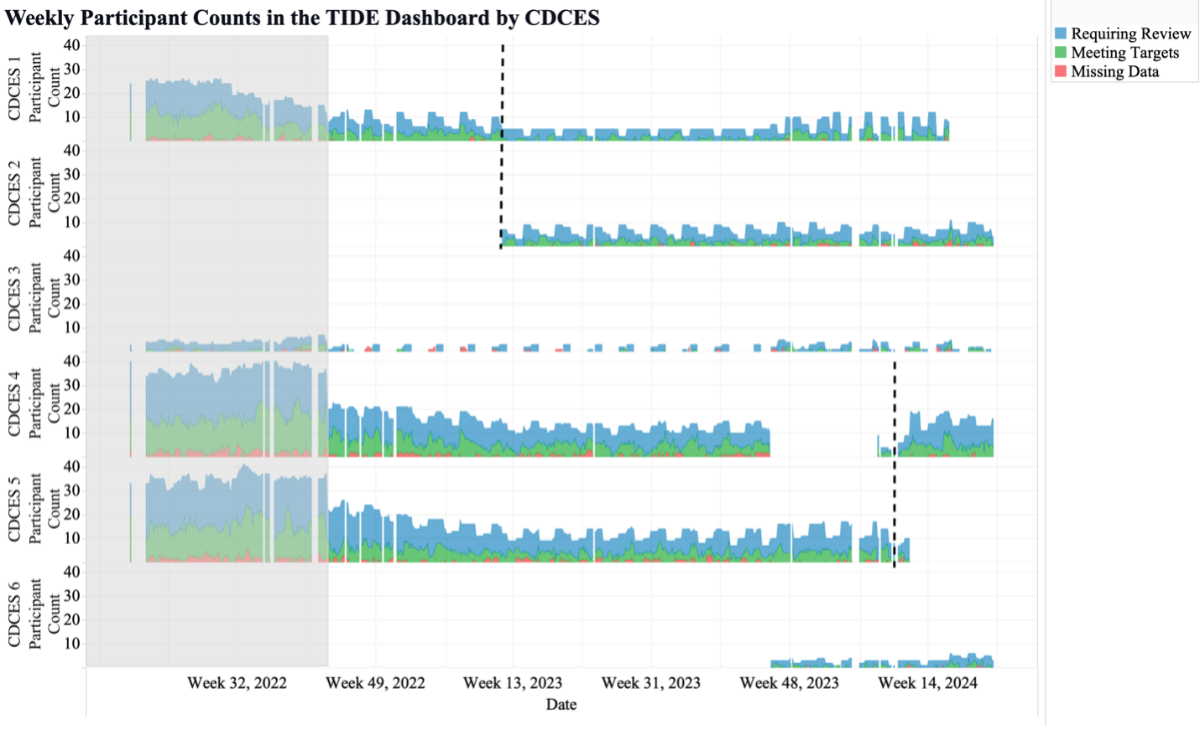
Weekly participant counts per Certified Diabetes Care and Education Specialist in the Timely Interventions for Diabetes Excellence dashboard, categorized by participants requiring review (blue), meeting targets (green), and with more than 50% missing data (red). The dashed lines mark the transitions of participants between CDCES team members. CDCES: Certified Diabetes Care and Education Specialist; TIDE: Timely Interventions for Diabetes Excellence.

The second metric was the number of participants shown in TIDE, categorized by each CDCES in the program. CDCES names are numbered to protect anonymity. This metric revealed significant differences in the number of participants assigned to each CDCES, as well as stability in the number of participants reviewed by each CDCES over time (Figure 3). Differences in the number of patients assigned to each CDCES can be attributed to the level of their patients’ complexity, their patients’ primary languages, and each CDCES’s time available to review participant data. This metric allowed for the monitoring of CDCES workload and identifying which CDCES team members may experience heavier participant workloads and require additional support, enabling workload balancing.

**Figure 4.**
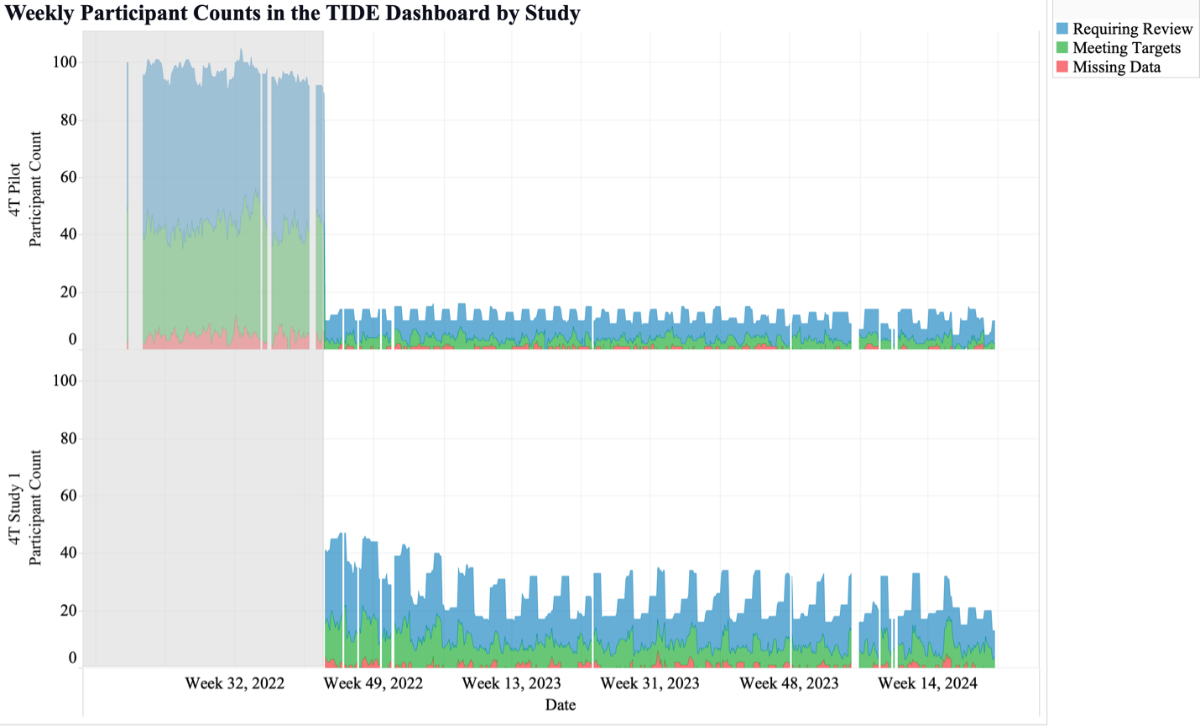
Weekly participant counts per study in the Timely Interventions for Diabetes Excellence dashboard, categorized by participants requiring review (blue), meeting targets (green), and with more than 50% missing data (red). TIDE: Timely Interventions for Diabetes Excellence.

The third metric was the weekly number of participants shown in TIDE per study. This metric may help clinics with several studies view the number of participants in the RPM program for each study (Figure 4). Alternatively, this metric may be used for tracking the number of patients within various populations, such as the number of patients who are within 1 year of T1D diagnosis, at high risk for hypoglycemia, pregnant, non-English speaking, recently hospitalized, diagnosed with certain medical conditions or with certain family histories, using specific tools to manage their health, or pediatric versus adult patients.

### Metrics and Visualizations for Patient Outcomes

**Figure 5.**
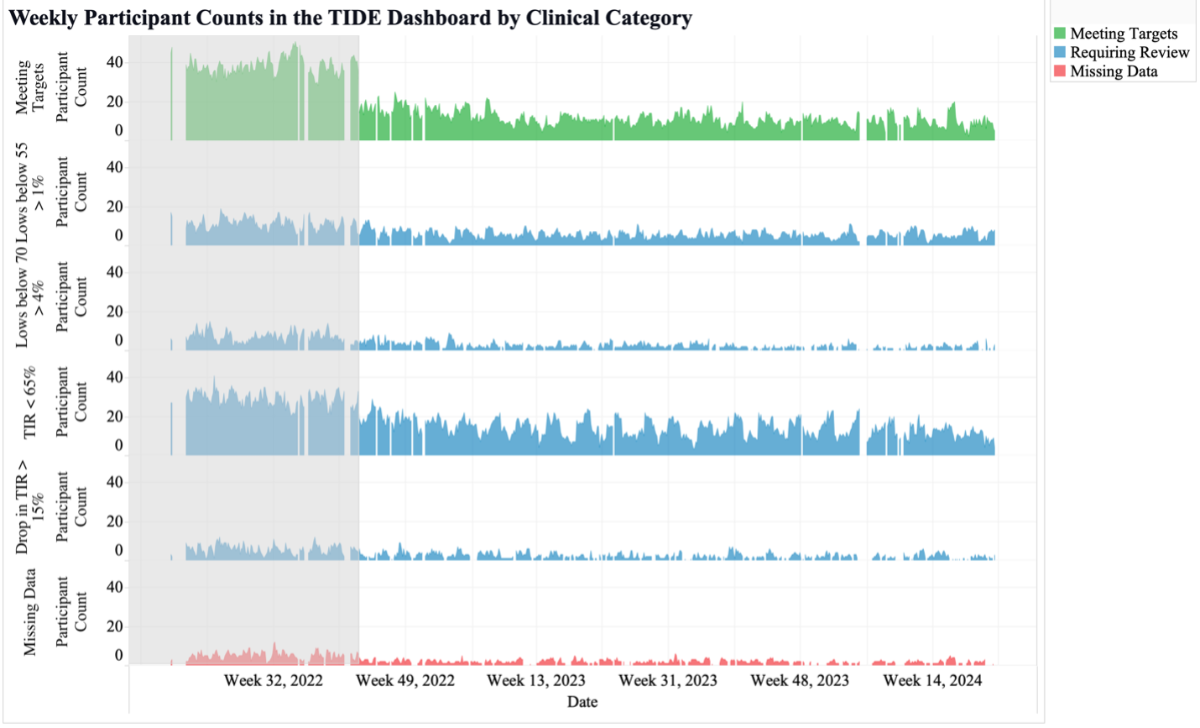
Weekly participant counts per clinical category in the Timely Interventions for Diabetes Excellence dashboard, categorized by participants requiring review (blue), meeting targets (green), and with more than 50% missing data (red). TIDE: Timely Interventions for Diabetes Excellence; TIR: time in range.

The fourth metric was the number of participants shown in TIDE per clinical category. This metric identified the level of variability in glucose management over time (Figure 5). This metric may help monitor participants’ data and identify areas of improvement or concern, allowing CDCES team members to adjust care plans and interventions. For example, an increase in the number of participants missing glucose data may prompt CDCES team members to educate patients on how to improve connectivity with their CGM and how to upload glucose data.

**Figure 6.**
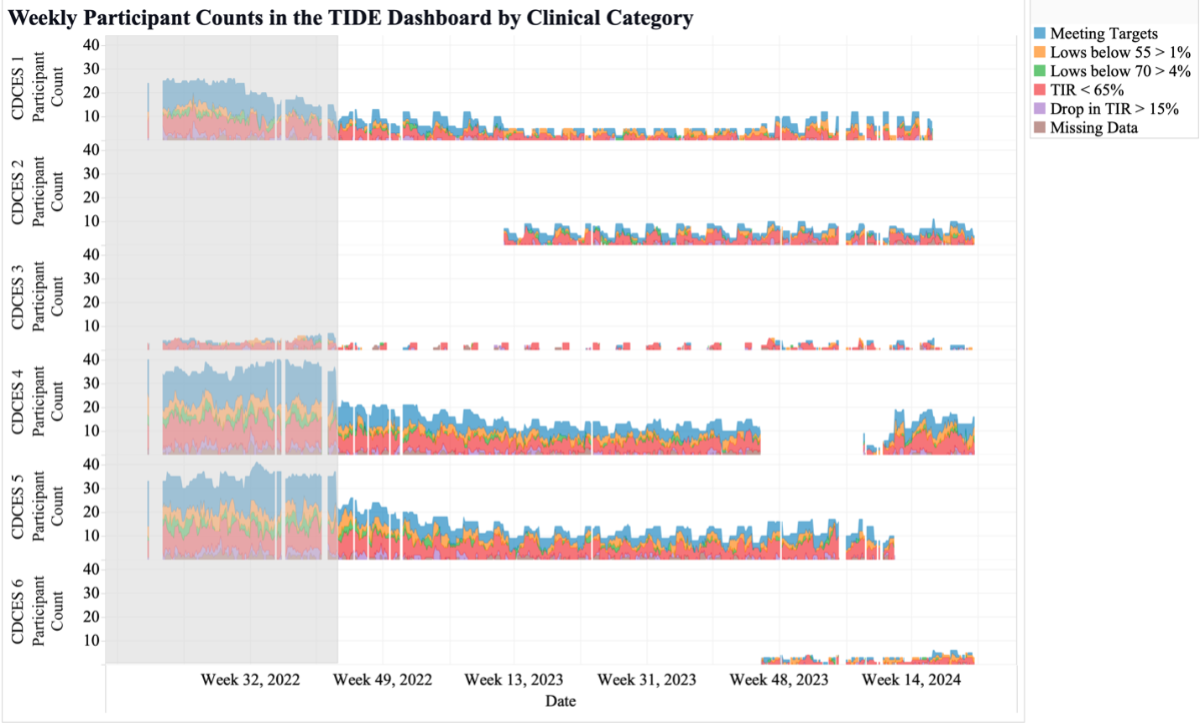
Weekly participant counts per Certified Diabetes Care and Education Specialist in the Timely Interventions for Diabetes Excellence dashboard, categorized by participants meeting targets (blue), spending more than 1% of continuous glucose monitoring readings below 55 mg/dL (orange), spending more than 4% continuous glucose monitoring readings below 70 mg/dL (green), spending less than 65% of continuous glucose monitoring time in range (70‐180 mg/dL; red), experiencing more than 15% point drop in time in range (purple), and missing more than 50% continuous glucose monitoring data (brown). CDCES: Certified Diabetes Care and Education Specialist; TIDE: Timely Interventions for Diabetes Excellence; TIR: time in range.

The fifth metric was the number of participants shown in TIDE per clinical category and reviewing CDCES. This metric identified the similarities and differences in glucose management for the participants reviewed by each CDCES (Figure 6). In our clinic, the metric revealed that all CDCES team members had similar proportions of participants in each clinical category (Figure 6). This metric can track how well each CDCES’s participants were meeting clinical consensus guidelines for CGM targets.

**Figure 7.**
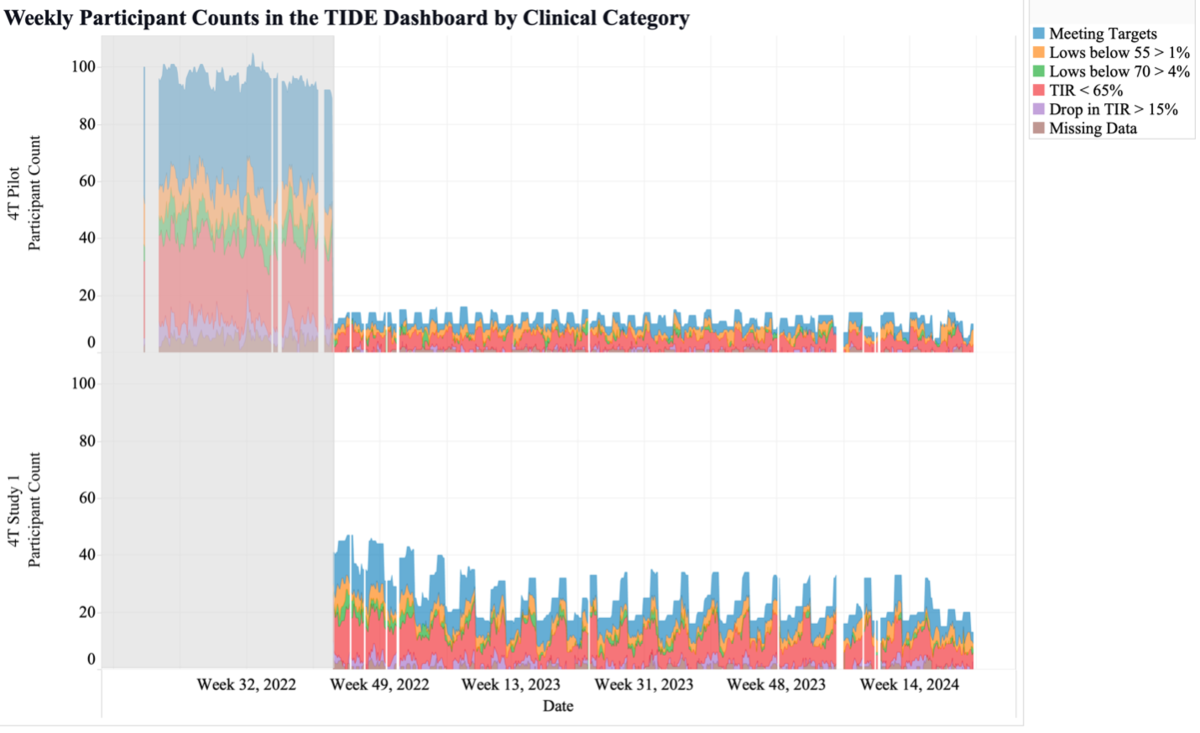
Weekly participant counts per study in the Timely Interventions for Diabetes Excellence dashboard, categorized by participants meeting targets (blue), spending more than 1% of continuous glucose monitoring readings below 55 mg/dL (orange), spending more than 4% continuous glucose monitoring readings below 70 mg/dL (green), spending less than 65% of continuous glucose monitoring time in range (70‐180 mg/dL; red), experiencing more than 15% point drop in time in range (purple), and missing more than 50% continuous glucose monitoring data (brown). CGM: continuous glucose monitoring; TIDE: Timely Interventions for Diabetes Excellence; TIR: time in range.

The sixth metric showed the weekly participant counts per study and clinical category. This metric may allow clinics to compare glucose management across studies or populations. This metric allowed the 4T team to identify variation in the numbers of participants in different studies and how often the groups were reviewed by CDCES team members (Figure 7). This provided insight into how well participants in different studies met clinical consensus glycemic targets. This metric facilitated easy comparison of health differences between different groups, study protocols (ie, Pilot vs Study 1), and clinical contact frequency (ie, weekly vs monthly contact).

### Metric and Visualization for Timeliness of Clinical Reviews

The seventh metric was originally the number of days since each participant’s most recent date of being displayed in TIDE. The patients shown were sorted from least to most recent display in TIDE and presented along with each participant’s patient ID, the CDCES that reviewed their data, and the study they were enrolled in. After review of this metric at a 4T leadership meeting, several alternative metrics and visualizations were proposed. The chosen visualization was a table comprising participants who had not been shown for more than 20 days and ranked in the top 25% of patients with the longest time since being shown. These restrictions focused on the patients most likely to benefit from review and limited the number of patients to be reviewed by the CDCES team. In addition to showcasing the days since their last appearance in TIDE, the table included each participant’s patient ID, the CDCES responsible for them, and the study they were enrolled in. If a participant had not been flagged in the RPM program in the last 20 days, this figure allowed the CDCES team to message or schedule a visit with participants, making sure no participants were inadvertently ignored. Similarly, this metric allowed clinical research coordinators to remove certain participants from studies that they were no longer enrolled in. For example, based on this metric, research coordinators checked if the participants who had not been shown in the TIDE dashboard for more than 50 days were still enrolled.

### Feedback From the Care Team

The engineering team developed and implemented 7 operational metrics mapped to 3 KPI domains and embedded them in a dashboard used in regular leadership meetings. Administrative, clinical, and research feedback indicated the framework helped monitor clinical workload, patient outcomes, and timeliness of care.

## Discussion

### Principal Findings

We developed a quantitative framework for monitoring clinical workload, patient glucose management, and timeliness of care for a whole-population T1D care model in which an algorithm analyzes CGM data to help direct care providers to patients with CGM metrics not meeting targets. Through a multiyear process of data analysis, data visualization, and feedback from different team members, we identified metrics that informed oversight and decision-making for the algorithm-enabled care model. This framework may be helpful for other pediatric T1D clinics seeking to optimize their RPM-based care strategies around algorithms that help direct clinician efforts. This framework enables clinics to transition from fixed-interval models to data-driven, responsive care models. Although this work focuses on RPM in a pediatric T1D population, the concepts may translate to adult T1D populations and other chronic health conditions in which algorithms support and guide clinical care [29-31].

The current framework complements, rather than duplicates or substitutes, the way clinical trials report measures of workload, efficiency, and PROs and family-reported outcomes. It complements those measures by aggregating them at the clinic and population level for standardized review on an ongoing basis. For example, in the 4T Study, explicit measures of efficiency measured as per-patient time have been reported in studies describing years of work, while the current KPIs are reviewed on a regular basis [2,32].

The metrics developed yielded valuable insights into the RPM program and may provide insights into health disparities. First, our glucose management metrics—the fourth, fifth, and sixth metrics—reinforce the safety of our RPM platform and the larger 4T Study. As shown in Figure 5, flags related to time below range were minimal, while a significantly larger share resulted from patients meeting all glycemic targets. This aligns with our published findings, which demonstrate improved HbA_1c_ and TIR, minimal TBR, and positive PROs [9,17,22,33].

Second, despite efforts to evenly distribute CDCES workloads, the number of individuals meeting criteria for review varied during certain review periods, which may have resulted in less time dedicated to those individuals. This finding prompted the care team to consider redistributing subsequent enrollees. Third, the number of participants shown in TIDE per clinical category and reviewing CDCES (the fifth metric) may be used as a starting point to compare the performance of CDCES team members. Meaningful comparisons will require significant additional controls and considerations, such as patient complexity, engagement, and level of access to health care. Fourth, this tool has the potential to identify positive outliers (CDCES team members whose patients achieve clinical targets) or to monitor the results of pilot interventions deployed by a subset of the CDCESs.

Finally, this metric may be used as a tool to investigate health disparities and the impact of patient-provider identity congruence on the relationship between CDCESs and their participants. By stratifying metrics by study group and individual CDCES, we can identify disparities in care delivery and patient outcomes. Previous work in the 4T Pilot Study has already reported similar improvement in HbA_1c_ for Hispanic and non-Hispanic youth, as well as for publicly and privately insured youth, but it may be valuable to continually monitor these comparisons (and possibly patient primary language) as part of the KPI framework [34]. The algorithmic prioritization of patients may also limit clinician bias and subjective decision-making, as well as prioritize high-risk patients who may otherwise be overlooked. In the future, we may investigate the disparity in usage of diabetes technologies, incongruence in clinician and patient perceptions of diabetes technologies, as well as how interest in and feasibility of diabetes technology programs for patients with public insurance change over time.

The ability to track clinical categories can help inform decisions related to clinical efficiency and care delivery. Guided by insights from the fifth metric, CDCES team members could stage interventions to improve glucose management or CGM wear time, compare our clinic’s TIR to other clinics, or observe how seasonal variations impact TIR.

The proposed framework is the first quantitative approach, of which we are aware, to monitor a CGM-based RPM program’s outcomes, process metrics, and workloads. Developing metrics that drive decision-making is critical, particularly in an era where algorithms can significantly influence patient care and clinical workload. In our clinic, the review of these algorithm-enabled KPIs is becoming standard practice, integral to monitoring and adjusting a care model in which algorithms reduce clinical workload.

### Recommendations to Implement KPIs and Metrics at a Clinic

KPI frameworks may be helpful for clinics that remotely access patient data, provide RPM-based care, and use algorithms to direct care delivery. Although our work focuses on RPM in a pediatric T1D population, these concepts may translate to an adult population, as well as other chronic health populations in which algorithms support clinical care. Using KPIs to monitor and adjust aspects of a care model may be particularly important for long-term interventions and large clinics or hospital systems [17].

To implement a KPI framework, the clinic or hospital might begin developing metrics by collaborating across multiple departments, potentially including internet technology specialists, data scientists, clinical research coordinators, clinicians, engineers, and quality improvement specialists. Clinics or hospitals may protect the time of or hire dedicated staff to implement the KPI framework and help with data analysis and visualization. We recommend that the internet technology team explore several platforms to host a dashboard for visualizing their metrics. Since large clinics may face scalability or data integration issues, it could be beneficial to partner with technology vendors or research collaborators. In a collaborative effort between Stanford’s 4T team and Tidepool (a diabetes technology non-profit), a new clinic-agnostic, turnkey solution called Tidepool-TIDE was made available to any clinic in the United States [35]. This may help simplify the KPI framework implementation process for other clinics and hospitals.

### Future Directions

Because of the success of our KPI framework, more metrics will be added in the future. We plan to add metrics related to CDCES time in TIDE, CDCES messaging activity, patient-clinician contact counts, PROs, and physical activity. These metrics have been researched as part of retrospective analyses, but it may be helpful to monitor them continuously as part of the KPI dashboard [2,7-9,18,22,24-26,36]. The above metrics have not yet been incorporated into the KPI framework because they are not integrated with the current workflow. We hope that the KPI framework supports the transition toward additional automation, such as using large language models to allow the platform to send some types of messages directly to the family [35,37]. The TIDE team continues to explore avenues for integration.

### Limitations

We acknowledge that the 4T Pilot and Study 1 populations are not representative of all other pediatric diabetes clinics, but were representative of the population diagnosed at our clinic [9,38]. This bias may limit the generalizability of our findings. Our study had high rates of early CGM adoption due to the design of the 4T intervention. Future implementation studies should include more representative samples outside of the context of broader research studies to validate the applicability of our framework across a wider range of clinical contexts. We also note that our study population is pediatric T1D, an autoimmune condition, in contrast to type 2 diabetes. T1D has the highest prevalence among non-Hispanic Whites, while type 2 diabetes has the highest prevalence among Native Americans and other non-White populations [39,40].

Although TIDE and the 4T Study have clearly improved patient outcomes and efficiency, we need to identify patients for whom repeated outreach is ineffective [2,8,9,17,21,22,25,26,32]. In future work, we will consider incorporating criteria to flag individuals who are repeatedly prioritized for review but are unreachable or do not seem to benefit from this prioritization. These patients may be switched from TIDE to a more traditional care model of quarterly reviews at clinic visits.

### Conclusion

Data from CGMs, insulin pumps, and automated insulin delivery systems play a growing role in the management of T1D. To best use the data, clinics increasingly turn to trusted, transparent, and user-friendly algorithms to translate patient data into insights that inform patient care. As the role of algorithms grows, the proposed framework may offer clinics a quantitative approach to monitor and potentially adjust the care delivery model.

## Supplementary material

10.2196/72676 Multimedia Appendix 1 Additional information on interviews, metric visualizations, and insights.

## Abbreviations

4T: Teamwork, Targets, Technology, and Tight Control
CDCES: Certified Diabetes Care and Education Specialist
CGM: continuous glucose monitor
EHR: electronic health record
HbA_1c_: hemoglobin A_1c_
KPI: key performance indicator
PRO: patient-reported outcome
REDCap: Research Electronic Data Capture
RPM: remote patient monitoring
T1D: type 1 diabetes
TBR: time below range
TIDE: Timely Interventions for Diabetes Excellence
TIR: time in range
