# Supplementary material for: A Quantitative Framework for Evaluating the Performance of Algorithm-Directed Whole-Population Remote Patient Monitoring: Tutorial for Type 1 Diabetes Care
Source: JMIR Diabetes. 2026 Mar 25;11:e72676. doi: 10.2196/72676 (PMC13016190; doi:10.2196/72676)
Supplement: Multimedia Appendix 1 [file diabetes-v11-e72676-s001.docx]

Multimedia Appendix 1

A Quantitative Framework for Evaluating the Performance of Algorithm-Directed Whole-Population Remote Patient Monitoring for Type 1 Diabetes Care

Section 1

Systematic interviews were conducted at biweekly TIDE meetings. Non-systematic interviews with individual clinicians and researchers followed up on questions from the systematic group interviews.

Interview questions (for each metric and visualization):

1. How do you interpret this metric?
2. What decisions does this metric drive? At what values does this metric drive decisions?
3. If the metric does not drive decisions, can it be modified to drive a decision? Or should it be discarded?
4. Is the visualization for the metric clear?

Section 2

For the visualizations of the first six metrics (of seven), we placed the week of load date on the x axis and the patient count on the y axis to make a line graph. We calculated the patient count by summing up the number of unique patient ID numbers that appeared in the TIDE dashboard each week.

Figure 2 (corresponding to the first metric) is composed of just the x and y axes, as well as a legend of three different patient statuses: Meeting Targets, Requiring Review, and Missing Data. Figures 3 and 4 feature the same legend and x and y axes, but they add another dimension. Figure 3 is “stacked” with a plot for each CDCES. Each patient is assigned to a CDCES, so the patient’s ID corresponds to a CDCES name. Figure 4 is also a “stacked” figure, this time with a plot for each study. Each patient ID corresponds to one study.

Figure 5 (corresponding to the fourth metric) has the week of load date on the x axis and the patient count on the y axis with different clinical categories having their own plots, making the figure appear stacked. This figure includes a legend of patient status. Figure 6 also has the week of load date on the x axis and the patient count on the y axis. Figure 6 is “stacked” with a plot for each CDCES, and the legend contains each clinical category (Meeting Targets, TBR level 2 >1%, TBR level 1 > 4%, TIR < 65%, Drop in TIR > 15%, and Missing Data). Figure 7 is the same as Figure 6, but each “stacked” plot now represents each study, not each CDCES.

We have not included a visualization for the seventh metric due to its sensitive and protected health information. The seventh metric shows the number of days since a participant was shown in the RPM dashboard. We display participant information only if the number of days since the participant has been shown in the TIDE dashboard exceeds 20 days and is in the highest quartile. This visualization is presented, along with additional information, in a table with four columns. The first column displays the number of days since each participant was shown in the TIDE dashboard; the second column shows the Patient ID; the third column shows the CDCES assigned to each patient; and the last column displays the study the patient enrolled in. We calculated the number of days since each participant was shown in the TIDE dashboard by using a filter to find the most recent date of each patient’s TIDE appearance. We then calculated the date difference between the date and today.

Section 3

Because of the KPI dashboard, we identified several key insights. As seen in Figure 2, the periodicity in the figure may be explained by the unequal number of participants assigned to each week. The repetitive patterns in the data were due to the cadence of CDCES reviews. For example, 4T Pilot initially reviewed participants weekly but switched to monthly after 1 year of diagnosis, which contributes to the large decrease in participants seen around week 40 of 2022.

Figure 3 captured the redistribution of participants from CDCES 1 to CDCES 2 in Week 12 of 2023. Similarly, this metric showed that CDCES 5 stopped reviewing data in Week 11 of 2024, and CDCES 4 took on the review of their participants (Figure 3).

As shown in Figure 4, the major drop in weekly participants in 2022 corresponded to the switch to the 4T Pilot long-term follow-up program, which reviewed participants monthly instead of weekly. The sharp decline in week 40 of 2022 occurred because all patients who had not yet reached a one-year duration in the 4T Study were moved to the long-term follow-up phase following the conclusion of the 4T Pilot.

As shown in Figure 7, the drop-off across all clinical categories before week 49 of 2022 occurred because the 4T Pilot ended, and not all participants chose to continue in the long-term follow-up program. Additionally, while the 4T Pilot reviewed patient data weekly, the long-term follow-up program did so monthly, resulting in fewer participants appearing in the TIDE dashboard. This decline is most apparent in the “Meeting Targets” category, as participants not meeting targets are prioritized and flagged for review over those who are meeting them.
